# Amelioration of Endothelial Dysfunction in Diabetes: Role of Takeda G Protein–Coupled Receptor 5

**DOI:** 10.3389/fphar.2021.637051

**Published:** 2021-04-28

**Authors:** Zhengyao Cai, Suxin Yuan, Yi Zhong, Li Deng, Jiafu Li, Xiaoqiu Tan, Jian Feng

**Affiliations:** ^1^ Department of Cardiology, The Affiliated Hospital of Southwest Medical University, Key Laboratory of Medical Electrophysiology, Ministry of Education and Medical Electrophysiological Key Laboratory of Sichuan Province, Institute of Cardiovascular Research, Southwest Medical University, Luzhou, China; ^2^ Department of Rheumatology, The Affiliated Hospital of Southwest Medical University, Luzhou, China

**Keywords:** diabetes mellitus, endothelial dysfunction, TGR5, endothelial nitric oxide synthase, inflammation, oxidative stress

## Abstract

Diabetes mellitus (DM) eventually leads to chronic vascular complications, resulting in cardiovascular diseases. DM-associated endothelial dysfunction (ED) plays an important role in the development of chronic vascular complications. Low endothelial nitric oxide synthase (eNOS) activity, inflammation, and oxidative stress all contribute to ED. The G protein–coupled receptor Takeda G protein–coupled receptor 5 (TGR5) is a membrane receptor for bile acids that plays an important role in the regulation of glucose metabolism. Recent studies have shown that TGR5 is involved in the regulation of various mediators of ED, which suggests that TGR5 may represent a target for the treatment of DM-associated ED. In this review, we summarize the principal mechanisms of DM-associated ED, then propose TGR5 as a novel therapeutic target on the basis of its mechanistic involvement, and suggest potential directions for future research.

## Introduction

Diabetes mellitus (DM) is a global public health problem that is associated with a high financial burden for healthcare systems. The progression of DM eventually involves the development of chronic vascular complications, which result in cardiovascular diseases. These cardiovascular complications are the main causes of death in diabetes patients around the world (Naveed et al., 2020). Although clinicians and researchers around the world continue to refine the management of DM, the increase in incidence of diabetic cardiovascular complications continues to outstrip improvements in their prevention and treatment.

There are many potential complications of DM, which have differing mechanisms. DM is considered to be a vascular disease in addition to a metabolic disease (Flyvbjerg, 2010; Arildsen et al., 2019) because vascular complications account for a relatively large proportion of diabetic complications and include diabetic cardiomyopathy, diabetic nephropathy, and diabetic peripheral neuropathy. Endothelial dysfunction (ED) is the initial vascular defect that develops in DM (Legeay et al., 2020; Lespagnol et al., 2020), and it is recognized to be an independent predictor of poor prognosis in patients with microvascular or macrovascular complications of DM (Wiggenhauser and Kroll, 2019; Villano et al., 2020). In this review, we focus on the ED that develops in diabetes, because impairment in endothelial function usually develops before related complications manifest clinically; by reducing ED, we can minimize target organ damage, and by identifying ED, we may diagnose DM in asymptomatic individuals. However, it should be pointed out that DM-related pathology does not always require ED; instead, epithelial cells may be directly affected, such as podocytes and tubules in the diabetic kidney (Pagtalunan et al., 1997).

In patients with DM, the risk of vascular disease has increased by two-to-four times in recent decades (Fox et al., 2004). Macrovascular and microvascular complications related to DM, such as coronary heart disease, stroke, peripheral arterial disease, diabetic retinopathy (DR), and kidney disease (Adler et al., 2003; Yau et al., 2012; Einarson et al., 2018), represent the main health burdens in patients with DM. To date, the management of diabetes has largely focused on the control of hyperglycemia (Triggle and Ding, 2014); however, because the rising burden of DM is mainly related to its vascular complications, it is also important to consider how to protect vascular function in these patients. In fact, although some classic hypoglycemic drugs have been shown to both ameliorate ED and hyperglycemia (Zelniker et al., 2019), there are few specific treatments for the ED associated with DM. Furthermore, in most patients with DM, the goal of controlling cardiovascular disease (CVD) risk factors is not achieved, because hypoglycemic therapy alone does not seem to reduce the incidence of large vessel-related outcomes (Gerstein et al., 2008), and many of the drugs have side effects. For example, metformin, which has been widely used as the first-line therapy for type 2 DM in recent years, can cause gastrointestinal irritation, and other hypoglycemic drugs can cause weight gain and accelerate the loss of pancreatic beta-cells (Maedler et al., 2005; Bennett et al., 2011; Stein et al., 2013; Meza et al., 2019). Therefore, the new frontier in the treatment of DM-associated ED is to identify a hypoglycemic agent that provides endothelial protection and ameliorates ED and is as free of side effects as possible.

Takeda G protein–coupled receptor 5 (TGR5) is a G protein–coupled receptor that is expressed in many organs and tissues, but is also widely expressed in almost all types of endothelial cells (ECs). Recent studies have shown that TGR5 agonists are beneficial in DM and TGR5 has become a promising target for its treatment (Pellicciari et al., 2009; Thomas et al., 2009; Briere et al., 2015). Therefore, in this review, we focus on ED and the role of TGR5 in DM, with the aim of collating evidence for this type of potential targeted therapy for DM.

### Diabetes and ED

The endothelium is a layer of squamous epithelial cells that lines the inner surface of vascular systems. ECs form a barrier between blood vessels and tissues, and control the flux of substances in and out of tissues. They provide a metabolic interface between blood and tissue and are therefore essential for the maintenance of vascular homeostasis (Galley and Webster, 2004). The inability of the endothelium to maintain vascular homeostasis is referred to as ED, which is a systemic pathological state that is characterized by alterations to the EC phenotype toward less vasodilatation, and pro-inflammatory and pro-thrombotic states. ED is induced by a number of factors, including turbulent blood flow, shear stress, hypoxia, aging, hyperglycemia, hypercholesterolemia, and hypertension (Gokce et al., 2002; Libby et al., 2002).

ED forms the basis of the chronic microvascular and macrovascular complications of DM. Microvascular complications include DR, nephropathy, and neuropathy, and macrovascular complications affect coronary and peripheral arteries, causing cardiovascular and cerebrovascular diseases and stroke (Suganya et al., 2016). In recent years, great progress has been made in the understanding of the mechanism of EC dysfunction during DM and the difference in its pathogenesis in patients with type 1 diabetes mellitus (T1DM) and type 2 diabetes mellitus (T2DM). In T1DM, uncontrolled hyperglycemia and low concentrations of endogenous insulin are the key defects that are involved in the pathogenesis of ED (Joshua et al., 2005), whereas in T2DM, dyslipidemia and insulin resistance play prominent roles (Hamilton et al., 2007). The metabolic milieu in diabetes, which involves hyperglycemia, insulin resistance, hyperinsulinemia, and obesity, induces a wide range of defects, and the principal pathophysiological processes that mediate the development of ED are oxidative stress, endoplasmic reticulum stress, and inflammation (Addabbo et al., 2009; Basha et al., 2012). Endothelial function is impaired in many tissues in diabetes and contributes to the impaired metabolic effects of insulin, as well as diabetic complications, and indicates that the endothelium is a potential target for the therapy of DM.

## Mechanism of Diabetic Damage to Endothelia

**FIGURE 1:**
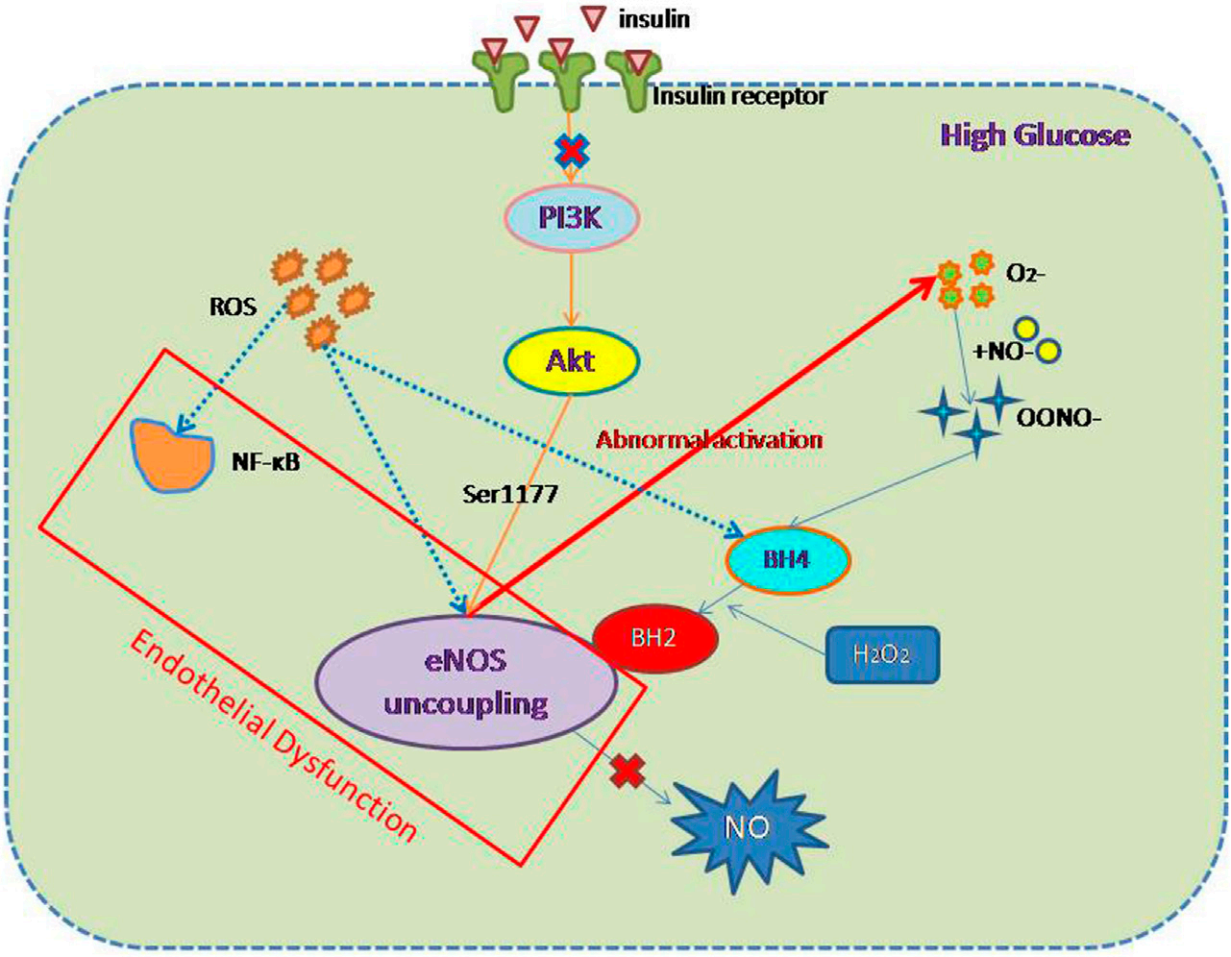
Mechanism of endothelial dysfunction associated with diabetes. In DM, cells exist in a high-glucose environment, but the etiology of ED associated with DM can be divided into two components: first, oxidative stress and inflammation; and second the uncoupling of eNOS and a reduction in NO production. The main mediators of oxidative stress and inflammation are ROS and NF-κB. The mechanism of eNOS decoupling involves (1) abnormal activation of eNOS, leading to a reduction in NO production; (2) inhibition of the PI3K/AKT/eNOS pathway as a component of insulin resistance; and (3) ROS activation, leading to a reduction in normal eNOS activation and an increase in the concentration of inhibitory BH2.

Furchgott and Zawadzki first described the importance of the intact endothelium for acetylcholine-induced vasodilatation in 1980 (Furchgott and Zawadzki, 1980). The mediator of acetylcholine-induced relaxation was originally designated as endothelium-derived relaxing factor but was eventually identified as NO generated from the amino acid l-arginine by the enzyme eNOS (Palmer et al., 1987; Moncada et al., 1988). eNOS is the main subtype of the enzyme that mediates the physiological generation of NO in ECs. Studies have shown that the reduction in NO production in diabetic patients is involved in the pathogenesis of ED (Förstermann and Münzel, 2006). However, the ED that develops alongside hyperglycemia is not only the result of the reduction in NO production by eNOS but also insulin resistance, oxidative stress, and inflammation (Figure 1). The mechanisms whereby each of these factors promote endothelial injury will be described individually below.

### eNOS and Endothelial Dysfunction

eNOS is the most important source of NO in ECs (Huang et al., 1995), and the NO generated in this way is crucial for endothelial function (Meza et al., 2019). Therefore, factors that can affect the expression of eNOS also affect the production of NO and can influence the development of ED. In a high-glucose environment, eNOS and endothelial function uncouple, and specifically, abnormal activation of eNOS produces O_2_^−^ instead of NO. This superoxide quickly combines with NO to form nitrogen-peroxynitrite (ONOO^−^), which is a highly active oxidant (Stuehr et al., 2001), and reduces the bioavailability NO. It is worth noting that the reaction between NO and superoxide occurs three times faster than the enzyme-catalyzed reduction by superoxide dismutase (Faraci and Didion, 2004), which implies that the cytoplasmic superoxide concentration is an important determinant of ED. In addition, the oxidation of tetrahydrobiopterin (BH_4_) to dihydrobiopterin (BH_2_) by ONOO− and H_2_O_2_ limits the availability of eNOS substrates and prevents the production of NO (Meza et al., 2019). BH_2_ cannot function as a cofactor for eNOS, but it competes with the active cofactor BH_4_. This change in eNOS status is referred to as “eNOS uncoupling” and plays an important role in the development of diabetes-associated ED and CVD (Förstermann and Münzel, 2006; Yang et al., 2009; Karbach et al., 2014).

Insulin resistance also has a significant effect on eNOS production. Under physiological conditions, the delivery of insulin to tissues involves ECs, which regulate capillary recruitment and glucose absorption (Kubota et al., 2011). A large number of previous studies have shown that in insulin resistance, adipose tissue and muscle glucose transporter 4 (GLUT-4) translocation are impaired, blood flow is reduced after a meal, EC function is impaired, and NO production is lower. Insulin signal transduction and glucose uptake are impaired (Kim et al., 2005), which implies that NO production is related to insulin signal transduction. Research by Ardilouze *et al.* showed that insulin upregulates NOS-dependent vascular activity, thereby increasing total muscle blood flow and capillary recruitment (Ardilouze et al., 2012). This is achieved by insulin binding to its receptor and activating the phosphatidylinositol 3-kinase (PI3K)/protein kinase B (AKT)/eNOS pathway in the endothelium. The phosphorylation of eNOS at Ser1177 by AKT increases the production of NO (Dimmeler et al., 1999), which results in vasodilation. Insulin resistance is a key defect in T2DM and is therefore also one of the causes of diabetes-related ED. Therefore, the amelioration of insulin resistance should also ameliorate ED.

Multiple lines of evidence have demonstrated an association between greater production of eNOS and an amelioration of diabetic ED. Claybaugh et al. (2014) showed that supplementation of l-arginine (LA), an NO precursor, increases the NO concentration by stimulating the production of eNOS. They found that the glomerular filtration rate of diabetic rats increased when they were administered LA and provided evidence that this effect may be the result of increases in eNOS expression and urinary cGMP, which leads to renal microvascular dilation and the amelioration of ED. Liu (Liu et al., 2014) and Krishnan (Krishnan et al., 2015) have also reached the same conclusion. However, it is worth mentioning that although these findings suggest that increasing the expression of eNOS can ameliorate DM-induced ED, there are some limitations to this approach. eNOS dysfunction is only one of the mechanisms that mediate diabetic ED, and even if an increase in eNOS expression improves endothelial function, hyperglycemia would remain and continue to have adverse effects on endothelia. Therefore, it is still necessary to rigorously prevent and treat DM in order to achieve the maximum impact on ED.

### Oxidative Stress and Inflammation

Both oxidative stress and inflammation interfere with insulin signaling (Wen and Duffy, 2017), leading to insulin resistance, DM, and related complications (Baig et al., 2020), and these processes are considered to be important components of the pathogenesis of DM. Persistent oxidative stress and the destruction of normal cellular redox homeostasis typically occur alongside chronic inflammation and the two are highly interrelated; therefore, we will discuss their impact on ECs in diabetes together (Mallard et al., 2020). Vascular ECs are a key site of metabolic dysregulation in diabetes (Khan and Chakrabarti, 2006). In particular, excessive oxidative stress results in the degradation of NO, which causes an imbalance in the effects of vasoconstrictors and vasodilators on the endothelium (Harrison, 1997; Behrendt and Ganz, 2002).

The effects of oxidative stress in ECs are mediated through NADPH oxidase, xanthine oxidase, aldehyde oxidase, and glucose oxidase, which are all enzymes involved in the generation of reactive oxygen species (ROS) (Suganya et al., 2016). ROS are the principal activators of diabetes-related vascular dysfunction (Brownlee, 2001; Brown and Griendling, 2015; Shah and Brownlee, 2016) and have their effects *via* a number of intracellular signal transduction pathways (El-Daly et al., 2018), through which they alter the phosphorylation and sensitization of eNOS, and also *via* the oxidation of BH_4_ to BH_2_. Thus, ROS can inhibit the production of NO by eNOS through two pathways, which can also form a vicious circle: competition between BH_2_ and BH_4_ leads to the dissociation of dimeric eNOS into monomers, which further increases the production of ROS (Wever et al., 1997; Stroes et al., 1998).

ROS also cause redox imbalance through several molecular mechanisms, such as by activating pro-inflammatory signaling pathways and increasing the secretion of pro-inflammatory cytokines (Yaribeygi et al., 2019), which also have deleterious effects on ECs. Previous studies have shown that in streptozotocin-induced diabetic rats, oxidative stress and the production of a series of inflammatory cytokines, including IL-1β, TNF-α, IL-6, and IL-17A, are significantly increased (Zhu et al., 2020), *via* nuclear factor kappa-B (NF-κB) (Park et al., 2006; Elmarakby and Sullivan, 2012). Therefore, ROS production and the NF-κB pathway represent targets for the treatment of ED.

### TGR5 May Represent a Novel Target for the Treatment of Diabetic Endothelial Injury

The pathogenesis of ED in DM involves oxidative stress, chronic inflammation, and low NO production. TGR5 is a G protein–coupled receptor that is present in many organs and tissues and is widely expressed in almost all types of EC. Recent studies have shown that TGR5 mediates beneficial effects in DM and regulates various molecules that mediate DM-associated ED (Pellicciari et al., 2009; Thomas et al., 2009; Briere et al., 2015).

### TGR5 and Diabetes

TGR5 (Gpbar1) is a G protein–coupled bile acid receptor that has been widely studied in recent years. It is expressed in various tissues and cell types, such as the gall bladder, intestine, and placenta (Kawamata et al., 2003; Keitel et al., 2009). The roles for TGR5 in regulating metabolic homeostasis have been well documented. Recent studies have shown that TGR5 agonists are beneficial in T2DM and it has become a promising target for the treatment of DM (Pellicciari et al., 2009; Thomas et al., 2009; Briere et al., 2015). Sato *et al.* found that activation of TGR5 using oleanolic acid reduced the serum glucose and insulin concentrations of high-fat-diet-fed mice (Sato et al., 2007). In addition, Thomas et al. showed that the activation of TGR5 has beneficial effects on metabolism that include resistance to weight gain and improved glucose homeostasis and insulin sensitivity (Thomas et al., 2009). Such improvements in glucose homeostasis and insulin sensitivity implicate TGR5 agonists as potential novel means of ameliorating DM-induced ED.

### TGR5 and eNOS

The role of the NO produced by eNOS activity in the maintenance of vascular health and function has been well described (Förstermann and Münzel, 2006). The reinstatement of eNOS activity to improve the bioavailability of NO represents a promising therapeutic strategy, aimed at reducing the incidences of the macrovascular and microvascular complications of DM. Recent studies have shown that TGR5 activation increases eNOS expression through two mechanisms.

First, TGR5 is expressed in ECs (Keitel et al., 2007; Kida et al., 2013). Studies have shown that the activation of TGR5 protects against diabetic nephropathy (Wang et al., 2018). TLCA, TCA, and TCDCA, which are TGR5 agonists, have all been shown to induce eNOS expression and Ser1177 phosphorylation of the enzyme, leading to an increase in NO production (Keitel et al., 2007; Fiorucci et al., 2009). And there are some researchers who have found that increase in eNOS expression is mediated *via* the “bile-salt-TGR5-cAMP pathway,” which involves binding to the cAMP response element of the human eNOS promoter (Keitel et al., 2007), and the increase of cAMP levels triggered by INT-777 is dose-dependent (Thomas et al., 2009). So the activation of TGR5 may protect against DM-associated ED by inducing an increase in eNOS activation through this pathway. But there is one thing we need to pay attention: Kida et al. (2013) found that TGR5 increased eNOS expression in vascular ECs in a dose-dependent manner *in vitro*, but care should be taken in extrapolating these results to the endothelium of intact animals, because culture methods cannot fully mimic the complex environment of these cells in blood vessels, nor the complexity of *in vivo* biochemical and biophysical regulatory mechanisms.

Second, glucagon-like peptide 1 (GLP-1) is an intestinal hormone that has been shown to improve endothelial function (Pratley and Gilbert, 2008; Kolka and Bergman, 2013), and the insulinotropic action of GLP-1 in pancreatic beta-cells has been shown to mediate the glucose lowering effect (Nauck et al., 1997). It has also been shown that GLP-1 is the target of TGR5 and that its secretion is TGR5-dependent (Harach et al., 2012; Briere et al., 2015). The GLP-1 receptor is expressed on ECs and is involved in eNOS activation through a PI3K-dependent pathway (Chai et al., 2012; Lim et al., 2017), which can ultimately be summarized as TGR5-GLP-1-PI3K-eNOS pathway. Further work should aim to clarify the function of this pathway, which may provide new therapeutic targets. Another study also suggests that stimulating effects of TGR5 agonists in the pancreas are mainly due to GLP-1 released from alpha-cells that acts in a paracrine manner on beta-cells (Kumar et al., 2016), improve mass and function of beta-cells in diabetic mouse models (Zheng et al., 2015), resulting in the increase of insulin levels, and then reduce the blood sugar, and at the same time, GLP-1 can protect endothelial function by “GLP-1-PI3K-eNOS” pathway. It is worth noting that the GLP-1 receptor agonists currently on the market do not increase the secretion of endogenous GLP-1, and therefore, they may not prevent certain local effects of disease progression. TGR5 is expressed in many organs and tissues but is principally expressed in the intestine and is found in enteroendocrine L cells, and stimulation of GLP-1 secretion mainly by intestinal L cells is likely to have certain advantages (Knop, 2010; Holst and McGill, 2012). Therefore, TGR5 activation may represent a means of stimulating GLP-1 secretion, as an alternative means of treating DM and ED caused by DM.

As discussed, activation of the bile-salt-TGR5-cAMP and TGR5-GLP-1-PI3K-eNOS pathways induces eNOS expression, which should protect against the ED induced by DM. However, eNOS supplementation does not always protect the endothelium. Krishnan et al. (2015) found that non-diabetic mice which overexpress eNOS show more superoxide production and poor vasorelaxation, and earlier studies by Ohashi (Ohashi et al., 1998) and Yamashita (Yamashita et al., 2000) showed that mice which overexpress eNOS resist the action of vasodilators. However, under specific pathological conditions, the overexpression of eNOS has been shown to be beneficial in specific organs, and we speculate that different disease states are associated with differing vascular phenotypes. Further studies are required to determine which particular disease states would benefit from therapeutic approaches involving eNOS activation.

### Effects of TGR5 Activation on Oxidative Stress and Inflammation

As described above, oxidative stress and inflammation are important mechanisms whereby DM leads to endothelial damage. The inhibition of ROS production or inflammatory signaling *via* the NF-κB pathway represents targets for the treatment of such ED. Previous studies have shown that the activation of TGR5 by INT-777 reduces oxidative stress in human podocytes exposed to a high-glucose environment (Wang et al., 2016). In addition, research by Deng *et al.* showed that the effect of INT-777 to reduce oxidative stress is achieved *via* a reduction in the production of ROS and apoptosis in cardiomyocytes exposed to a high-glucose environment (Li et al., 2018; Deng et al., 2019). These studies suggest that TGR5 may ameliorate the DM induced ED by inhibiting the production of ROS. Meanwhile, previous studies have shown that the activation of TGR5 has anti-inflammatory effect on many kind of inflammations through NF-κB signaling pathway. There is evidence that TGR5-induced NO production inhibits the expression of adhesion molecules and the adherence of monocytes, thereby inhibiting NF-κB activity in ECs (Kida et al., 2013). Guo et al. (2015) also found that TGR5 became a negative regulator of gastric inflammation by antagonizing the NF-κB signaling pathway. And in macrophages, activation of TGR5 inhibited NF-κB–mediated inflammatory cytokine production (Pols et al., 2011). Moreover, the study of Wang et al. (2011) found that liver necrosis and inflammation were more severe in TGR5 knockout mice than in wild-type mice in an LPS-induced inflammation model. Therefore, the anti-inflammatory properties of TGR5 may be one of the mechanisms to improve ED caused by DM.

## The Identification of a Novel TGR5 Activator for the Treatment of Diabetes-Associated Endothelial Dysfunction

Low eNOS expression, inflammation, and oxidative stress are the principal mechanisms of ED in DM, and the means whereby conventional hypoglycemic drugs protect against ED mainly involve antagonism of these mechanisms (Lu et al., 2019). Many studies have shown that TGR5 plays an important role in DM, inflammation, and oxidative stress, and its agonists may represent novel potential drug substances for the treatment of metabolic and inflammatory disorders (Pols et al., 2011; Lo et al., 2017; Lyu et al., 2019). Therefore, it is important to study the mechanisms whereby TGR5 agonists ameliorate DM-associated ED, to further understand their potential.

Although the use of TGR5 agonists for the treatment of ED in DM holds great promise, a series of questions remain to be resolved. First, TGR5 is expressed in many organs, which may imply that an agonist would be prone to side effects in other organs. For example, Poole et al. (2010) found that the activation of TGR5 reduces gastric and intestinal motility, which can cause nausea and constipation. In addition, the activation of TGR5 delays bile emptying, which was shown to increase the incidence of gallstones over that of TGR5 knockout mice (Keitel et al., 2009; Li et al., 2011). Furthermore, the activation of TGR5 induces hyperexcitability in dorsal root neurons, which results in an itching sensation (Alemi et al., 2013). Finally, there is some *in vitro* evidence to suggest that TGR5 agonists may induce cancer cell proliferation (Hong et al., 2010; Casaburi et al., 2012; Cao et al., 2013).

Second, even in the same organ, the effects of TGR5 may differ, because of the existence of various distinct binding sites. For example, TGR5 is expressed in the primary cilia of cholangiocytes, where it is coupled to G-α and inhibits cell proliferation, but it is also expressed on the apical plasma membrane, where it is also coupled to G-α proteins but initiates cell proliferation (Masyuk et al., 2013).

Third, TGR5 binds a wide range of ligands, including endogenous and synthetic bile acids and neurosteroids, and also synthetic agonists with a nonsteroidal core (Sato et al., 2008; Martin et al., 2013). Although it is activated by all the known bile acids, the potency of everyone differs from other and depends on the hydrophobicity of its bilane scaffold, and this is reflected in differing effects on the target organ (Sato et al., 2008; Keitel et al., 2020).

Fourth, differing concentrations of TGR5 agonists may have different outcomes. For example, compound 18, a new type of TGR5 agonist, has a significant effect on biliary secretion at a dose as low as 3 mg/kg, whereas doses of compound 18 above 30 mg/kg have a significant effect on GLP-1 secretion (Briere et al., 2015).

Finally, even if an TGR5 agonist is used at the same dose, the results obtained in animal models and humans differ, because of the biological differences between species (Hodge et al., 2013). Therefore, for these reasons, the appropriate choice of TGR5 agonist is critical for its usefulness for the treatment of ED in diabetes.

Nevertheless, in recent years, substantial efforts have been made to identify suitable TGR5 agonists, and there have been some promising results. For example, Zheng et al. (2015) studied the effects of the novel TGR5 agonist WB403 in diabetic mice, and they found that WB403 is not associated with side effects in the gallbladder. Also, in Shan-yao MA et al.'s study, they designed a novel TGR5 agonist called OL3, which combined linagliptin, a DPP-4 inhibitor, with MN6, a novel TGR5 agonist, and they found that OL3 can also lower blood glucose levels without causing gallbladder filling in mice too (Ma et al., 2016); both of the two TGR5 agonists are at a low systemic concentration, and furthermore, WB403’s most significant effects occur in the intestine, which offers new ideas for the design of the best TGR5 agonist for ED caused by DM. And a recently published study also showed that the activation of TGR5 slows the progression of DR in diabetic rats (Zhu et al., 2020), which also confirms that the activation of TGR5 can reduce the ED associated with DM. Thus, although there are many obstacles to the development of TGR5 agonists for the treatment of DM-associated ED, some progress has been made. Nowadays, many researchers found different novel TGR5 agonists which have low side effects (Duan et al., 2015; Finn et al., 2019), future studies should be directed toward the pick of the best TGR5 agonist and evaluation of the safety of TGR5 agonists in both animal study and clinical study. In summary, in order to identify the most appropriate TGR5 agonist, the following issues should be addressed: (1) the biochemical properties of ligands that dictate their distribution and metabolism, such that ligands bind to TGR5 to achieve the target; (2) the design of ligands to render their downstream effects more specifically; and (3) tissue-specific targeting of TGR5 for activation or inhibition.

## Discussion

**FIGURE 2:**
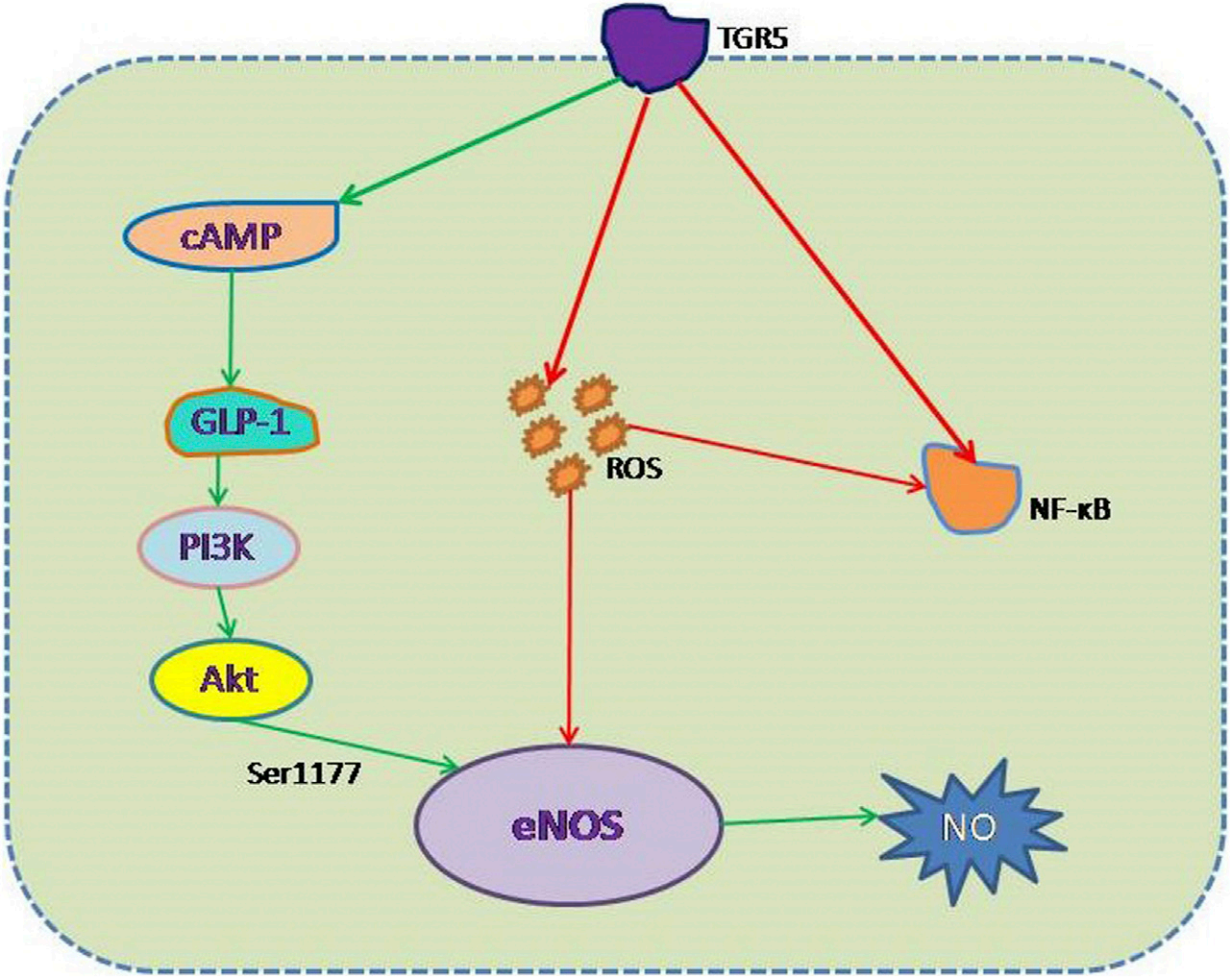
Mechanism whereby TGR5 protects against high-glucose-induced endothelial damage. (1) Negative effect (red line): inhibition of the generation of ROS and the activation of NF-κB, thereby reducing eNOS decoupling and NF-κB–mediated ED. (2) Positive effect (green line): increase in eNOS expression *via* the TGR5-GLP-1-PI3K-eNOS pathway, which mimics GLP-1 action.

The high prevalence of and numerous risk factors for CVD make it the leading cause of death in the world (Mathers and Loncar, 2006). However, many CVD patients also have DM, which predisposes toward CVD because it is associated with ED (Sun et al., 2019). Hyperglycemia in patients with DM is the principal risk factor for ED-mediated vascular complications, but DM can cause ED through a variety of mechanisms. Low eNOS expression, oxidative stress, and inflammation all cause ED in DM. Because blood from the intestines and the liver is dispersed throughout the body, the vascular endothelium is continuously exposed to bile acids. Therefore, bile acid signaling may affect the physiological functions of ECs. TGR5 is a bile acid receptor that has attracted a lot of attention in recent studies, and studies have shown that its activation has a beneficial effect in DM. This effect of TGR5 activation is mediated not only through a reduction in blood glucose, but also by protecting EC function by increasing eNOS expression, promoting GLP-1 secretion, reducing insulin resistance, and reducing oxidative stress and inflammation (Figure 2). All of these outcomes imply that the activation of TGR5 may represent a therapeutic target for ED in DM. The identification of the optimal TGR5 agonist represents the next research goal.
